# Supplementary material for: Applications for Circulating Cell-Free DNA in Oral Squamous Cell Carcinoma: A Non-Invasive Approach for Detecting Structural Variants, Fusions, and Oncoviruses
Source: Cancers (Basel). 2025 Jun 6;17(12):1901. doi: 10.3390/cancers17121901 (PMC12190202; doi:10.3390/cancers17121901)
Supplement: Supplementary file 1 [file cancers-17-01901-s001.zip › Supplementary information.pdf]

Supplementary information:

Table S1A: Demographic information of healthy vs OSCC patients

30 OSCC patients and 25 healthy individuals were considered in our studies. The average cfDNA of healthy individuals were at 1.04ng/ml while OSCC patients were 23.72ng/ml.

Table S1B: Details of OSCC patients:

Different parameters like smoking status, alcohol consumptions, and clinical parameters like tumor site, pre-existing diseases, p53, Ki67 and keratinization status are mentioned.

Table S2: Fusion primers

Primer sequence for *TRMO-TRNT1* gene fusion.

Table S3: PCR conditions

PCR conditions for fusion detection in OSCC samples.

Figure S1: Bioanalyzer graph of cfDNA size of OSCC patients: The arrow mark in black shows peak of cfDNA size in bioanalyzer graph

Figure S2: Fusion sequence: the purple sequence indicates sequence of TRMO, red sequence indicates junction and blue sequence indicates TRNT1.

Figure S3: Fusion validation in other OSCC samples: A. Sanger sequencing of the PCR products confirmed the fusion junction in our samples with results from the ChiTaRS fusion database. B. Agarose gel image of fusion (highlighted by the red box) identification in other patients' cfDNA after PCR.

Figure S4: Coding potential of the fusion sequence. A. The CPAT score suggests that fusion to be non-coding. B. The CNIT result was also used to validate the coding potential and likewise suggests that the fusion gene is a non-protein coding sequence.

Table S1A: Demographic parameters of healthy vs OSCC patients

Details	Healthy	OSCC Patients
**Gender**	Male: 10 (40%)<br>Female: 15 (60%)	Male: 14 (47%)<br>Female: 16 (53%)
**Age (mean)**	52.90	58.25
**cfDNA concentration**	Mean 1.04<br>Median 1.2	Mean 23.72<br>Median 22.83

Table S1B: Details of OSCC patients

Parameters	OSCC Patients
**Smoking status**	Current – 7<br>Past – 4<br>Never – 19
**Alcohol abuse**	NA
**Diabetes Mellitus**	Present: 9 (30%)<br>Absent: 21 (70%)
**IHD**	Present: 5 (16%)<br>Absent: 25 (84%)
**S/P CVA**	Present: 3 (10%)<br>Absent: 27 (90%)
**Other past malignancy**	Present: 7 (23%)<br>Absent: 23 (77%)
**Primary site**	Tongue – 20 (67%)<br>Alveolar ridge – 8 (27%)<br>FOM – 1 (3%)<br>RMT – 1 (3%)
**Ki67**	Positive: 16 (53%)
**P53**	Positive: 14 (47%)
**Keratinization**	Present: 20 (67%)
**P16**	Positive: 3 (10%)

Table S2: Fusion primers:

Forward	CTCCTGAGAACCACCAGACA
Reverse	GCTGCACCCCACTAATGTGT

Table S3: PCR conditions:

Initial denaturation	95 ^{o}C	2 mins	
Denaturation	95 ^{o}C	25 sec	28 cycles
Annealing	53°C	35 sec	
Extension	72 ^{o}C	60 sec	
Final Extension	72 ^{o}C	5 mins	
Hold	4°C	∞	

Figure S1

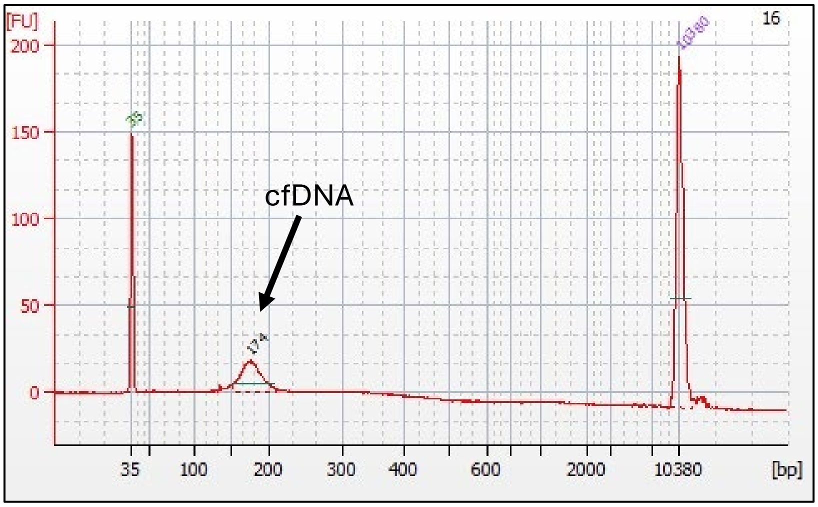

[FU]
200
150
100
50
0
35
174
10380
cfDNA
16
35
100
200
300
400
600
2000
10380
[bp]

Figure S2

GGGGCTTGGAGGAGCCGGGGCCTCGGCCTACAGCGACCCCGTGCGGCTGCGTGAAGCCGGCTCTGGAGACAG
GGAATCTTTTAACTGAGCCAGTCGGCTACTTGGAATCTTGTTTCTCGGCCAAGAATGGTACTCCAAGACAGCCAT
CCATTTGTAGCTATTCTCGAGCCTGTTTGAGGATTAGAAAGAGGATCTTTAATAATCCTGAACATTCCTTGATGG
GCCTAGAACAGTTTTCTCATGTTTGGATTTTGTTTGTTTTTCACAAAAACGGTCATTTGAGCTGTAAGGCAAAAGT
GCAGCCTCCTAGGCTGAATGGTGCAAAGACTGGAGTTTTTTCCACAAGGAGCCCTCATCGTCCCAATGCAATAG
GACTGACCCTGGCCAAGCTGGAAAAGGTAGAAGGTAACCCATTTCATTTCTACTTTTACCTTTTTCCTCACCCAAA
AAAGCCTTATAAAATAAACAGCATTCACCACTGGCTGTTTATTCAGACGAGTACCAATGATGTTAACTCCTGAGA
ACCACCAGACATTGAAGAAAATACAATGATTGGGGGGAGGGATAGTATTGGGAGATATACCTAATGCTAGATG
ACACATTAGTGGGGTGCAGCGCACCAGCATGGCACATGTATACCTATGGTAACTAACCCTGCACAATGGTGCAC
ATGTTACCCCTAAAACCTTAGAGGTATAATTAAAAAAAAACACAAAAAAGAAGT

Figure S3

A

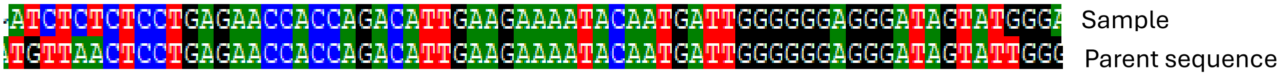

ATCTCTCTCCTGAGAACCACCAGACATTGAAGAAAATACAATGATTGGGGGGAGGGATAGTATGGGA
Sample
ATGTTAACTCCTGAGAACCACCAGACATTGAAGAAAATACAATGATTGGGGGGAGGGATAGTATTGGG
Parent sequence

B

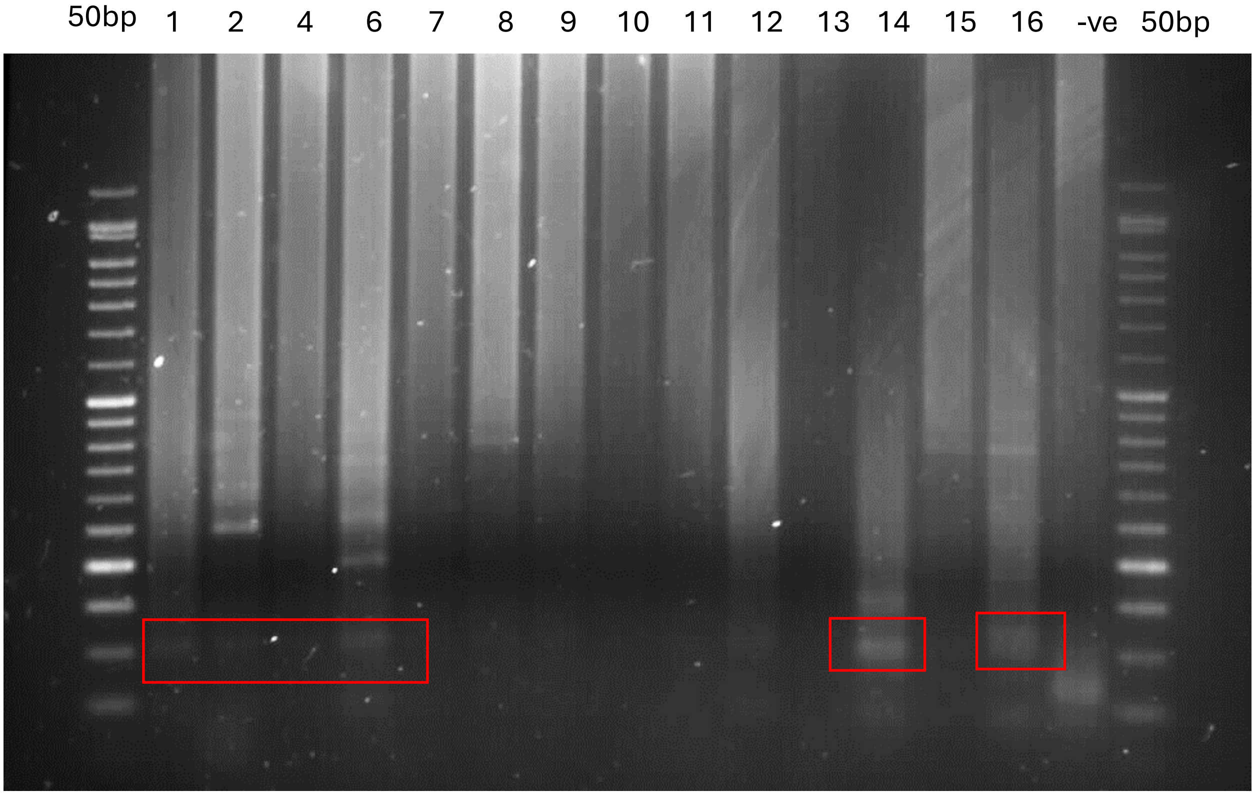

50bp
1
2
4
6
7
8
9
10
11
12
13
14
15
16
-ve
50bp

Figure S4

A

CPAT
Coding Potential Assessment Tool

Calculator User Guide Feedback Source Code

Result for species name : hg19 with job ID :1656560183

Data ID	Sequence Name	RNA Size	ORF Size	Ficket Score	Hexamer Score	Coding Probability	Coding Label
0	HG19	724	303	0.8793	-0.10848869517	0.05633330577838	no

This job has been stored with the job ID
Download Table in tab delimetered file (.txt)

For suggestions, comments or queries about this website,
please leave your feedback through Feedback.
Copyright © 2012. All rights reserved.

BCM
Baylor College of Medicine
MAYO CLINIC

B

CNIT Score Detail Plot

Red line represents the correct transcriptional reading frame and other five lines (blue or green) represent other five reading frames. Green line indicates the distribution of the coverage (the right y-axis) of the MLCDS region for each transcript across the normalized length.

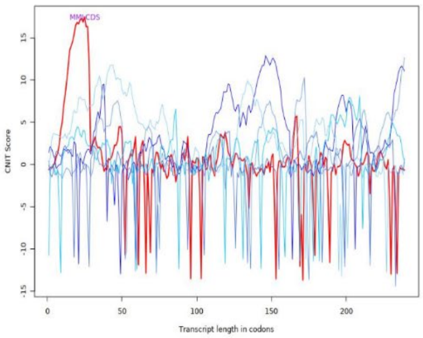

MLCDS: The most-like CDS region
Codon length: The total length of the identified sequence converted to codons length (the identified sequence length/3).

Blast the sequence in NONCODE Run
